# Development and Application of a Simple Plaque Assay for the Human Malaria Parasite *Plasmodium falciparum*

**DOI:** 10.1371/journal.pone.0157873

**Published:** 2016-06-22

**Authors:** James A. Thomas, Christine R. Collins, Sujaan Das, Fiona Hackett, Arnault Graindorge, Donald Bell, Edgar Deu, Michael J. Blackman

**Affiliations:** 1 The Francis Crick Institute, Mill Hill Laboratory, Mill Hill, London, NW7 1AA, United Kingdom; 2 Department of Microbiology & Molecular Medicine, University of Geneva, Rue Michel-Servet 1, CH-1211, Geneva 4, Switzerland; 3 Department of Pathogen Molecular Biology, London School of Hygiene and Tropical Medicine, London, WC1E 7HT, United Kingdom; Bernhard Nocht Institute for Tropical Medicine, GERMANY

## Abstract

Malaria is caused by an obligate intracellular protozoan parasite that replicates within and destroys erythrocytes. Asexual blood stages of the causative agent of the most virulent form of human malaria, *Plasmodium falciparum*, can be cultivated indefinitely *in vitro* in human erythrocytes, facilitating experimental analysis of parasite cell biology, biochemistry and genetics. However, efforts to improve understanding of the basic biology of this important pathogen and to develop urgently required new antimalarial drugs and vaccines, suffer from a paucity of basic research tools. This includes a simple means of quantifying the effects of drugs, antibodies and gene modifications on parasite fitness and replication rates. Here we describe the development and validation of an extremely simple, robust plaque assay that can be used to visualise parasite replication and resulting host erythrocyte destruction at the level of clonal parasite populations. We demonstrate applications of the plaque assay by using it for the phenotypic characterisation of two *P*. *falciparum* conditional mutants displaying reduced fitness *in vitro*.

## Introduction

As one of the most important human pathogens, much research on the human malaria parasite *Plasmodium falciparum* focuses on the identification and characterisation of drug targets and/or an improved understanding of how host immune responses interfere with parasite replication and associated pathology. During the clinically relevant asexual blood stage of the parasite lifecycle, merozoites invade host erythrocytes where they divide within a parasitophorous vacuole to produce 16–20 daughter merozoites. These are then released from the erythrocyte, completely destroying it in the process. In certain regards the parasite blood-stage lifecycle therefore mimics a viral lytic cycle, in that destruction of each host cell allows the release of multiple invasive forms which go on to invade and destroy further host cells, amplifying the pathogen population. For many viruses, this lytic cell cycle has long been exploited in *in vitro* assays in which the concentration of infectious viral particles in a sample can be determined by microscopic visualisation of destruction of host cells following their infection by suitably titrated aliquots of virus. First described for animal viruses by Dulbecco and Vogt in 1953 [1], the assay protocol usually involves limiting diffusive dispersion of the released viral particles through the use of semi-solid media in order to achieve discrete, highly localised regions of host cell monolayer destruction called plaques. The cell monolayers are finally stained to visualise the plaques. Because of their simplicity and broad applicability, plaque assays are amongst the most valuable and widely-used tools in viral research, allowing facile quantitation of the effects on viral replication of environmental conditions, drugs, antibodies and genetic manipulation, and simplifying isolation of viral clones. Plaque assays have also been developed for other intracellular pathogens, including several bacterial species [2] and even protozoan organisms related to the malaria parasite, notably *Toxoplasma gondii* which readily infects most nucleated mammalian cells and so can be cultured in adherent fibroblast monolayers [3]. In contrast, blood stages of *P*. *falciparum* and other *Plasmodium* species pathogenic to humans replicate exclusively in erythrocytes (or reticulocytes), which are not normally adherent. Plaque assays developed for *Plasmodium* have therefore used monolayers of erythrocytes adhered to the base of plastic tissue culture wells using concanavalin A [4, 5], Cell-Tak [6], or anti-Rhesus D antibodies plus protein L [7], with plaque formation being visualised using either Giemsa staining of fixed monolayers or immunofluorescence. Such assays were key to the success of elegant pioneering experiments demonstrating the phenomenon in which all the merozoite offspring of a single infected erythrocyte are committed to either continuation of the asexual life cycle or transformation into either male or female forms of the sexual stages (gametocytes) responsible for transmission to the mosquito vector [4, 5, 7]. However, due to the single-cell-thick nature of the adherent erythrocyte monolayers produced by these methods and the need for fixation and staining to visualise the plaques, the assays are unsuitable for routine quantitation of malaria parasite growth rates. Here we describe the optimisation and application of an extremely simple plaque assay that we expect will become an attractive and widely used addition to the available repertoire of malaria research tools.

## Results

### *P*. *falciparum* Growth in Static Erythrocyte Cultures Produces Plaques

**Fig 1:**
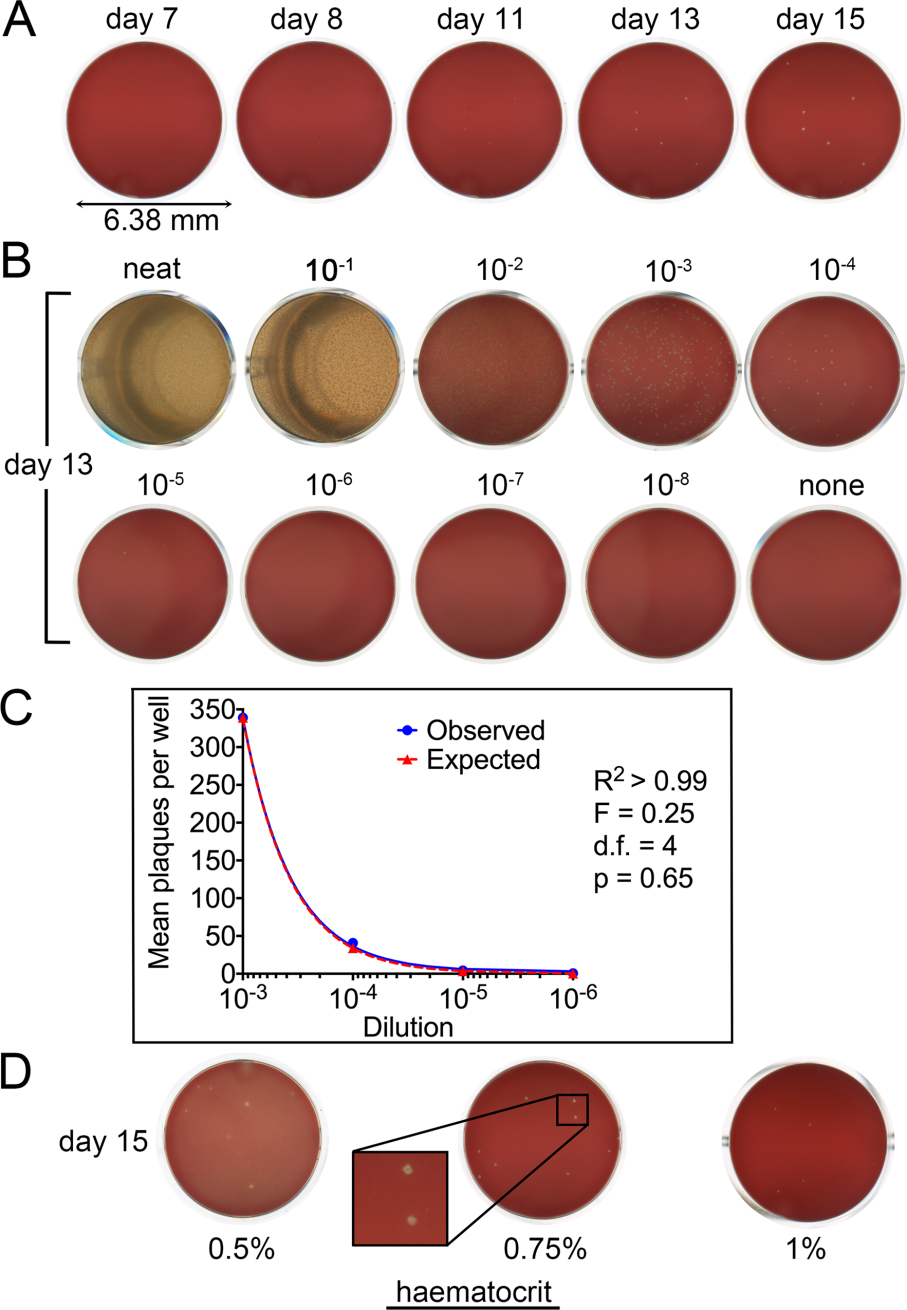
Formation of plaques in static *P*. *falciparum* asexual blood stage microplate cultures. (A) Time-dependent evolution of plaques. Scanned images (RGB) of a single microplate well (diameter 6.38 mm) taken at the indicated times following the introduction of a low parasitaemia *P*. *falciparum* 3D7 culture (200 μl per well, 0.75% haematocrit, starting parasitaemia ~0.00004%, corresponding to ~6 infected cells per well). Plaques first became detectable by light microscopy or high resolution scanning at day 8. (B) Plaque density correlates with starting parasitaemia. Shown are microplate wells containing 10-fold serial dilutions (indicated) of a *P*. *falciparum* culture (0.75% haematocrit, starting parasitaemia 13%). The wells were imaged on day 13 following initiation of the culture. By this point, at the highest parasite densities (neat and 10^−1^ dilution) the erythrocyte layer was completely destroyed, whilst discrete plaques were visible at the lower parasite densities. Total numbers of plaques in each set of 6 replicate wells of the 10^−3^–10^−8^ dilutions (replicate wells are not shown) was 2,034 (10^−3^), 245 (10^−4^), 26 (10^−5^), 4 (10^−6^), and 0 plaques (10^−7^ and 10^−8^). No plaques were ever detected in the complete absence of parasites (none). (C) Linear regression by analysis of covariance (ANCOVA) indicating a strong linear inverse correlation between dilution and plaque density in the wells containing the 10^−3^–10^−6^ parasite dilutions. A plot of observed mean plaque frequency against dilution (blue) from the experiment shown in (B) is shown alongside a plot of the plaque frequencies expected if there is a linear inverse correlation between plaque number and dilution (red). Values of the statistical data (R^2^, F statistic, number of degrees of freedom and p value) are shown. (D) Optimisation of haematocrit conditions. Microplate wells containing cultures at ~0.00004% parasitaemia at the indicated haematocrits, imaged on day 15. Whilst plaque formation was easily detected in the 0.75% haematocrit wells, they were much more difficult to detect at higher haematocrit, whilst at lower haematocrit values the plaques were typically more diffuse with signs of erythrocyte lysis. Inset, zoomed region of the 0.75% haematocrit erythrocyte layer, showing the discrete nature of the plaques.

In initial work, asexual blood-stage cultures of *P*. *falciparum* (clone 3D7) were dispensed in complete medium into the central 60 wells of flat-bottomed 96-well microplates and incubated undisturbed (without replacing the medium or disturbing the erythrocyte layers) at 37°C in sealed, humidified gassed chambers, monitoring by daily examination with an inverted light microscope. This revealed the gradual appearance and expansion of translucent, roughly circular discontinuities or apparent zones of clearance in the otherwise homogeneous erythrocyte layer coating the base of each well (Fig 1A). These discontinuities are henceforth referred to as plaques. Importantly, plaque formation was easily detected and recorded without opening the plates using a high resolution flatbed digital scanner (top-down transmission light mode, 4,800 dpi), avoiding the need for frequent and laborious microscopic examination of individual wells and also allowing simple recording and documentation of the plate images. To formally establish whether plaque formation was a result of parasite replication, synchronous parasite cultures (~10% parasitaemia, 0.75% haematocrit) were serially diluted into a suspension of fresh uninfected erythrocytes (0.75% haematocrit) in complete medium and aliquots of each dilution dispensed across a microplate. Monitoring of plaque formation in these plates showed that the mean frequency of plaques at any given dilution correlated linearly with the starting parasitaemia in each well (Fig 1B and 1C and S1 Fig). No plaques formed in the absence of parasites. These results suggested that, so long as the erythrocyte cultures remained static and undisturbed, parasite growth with concomitant host cell lysis was capable of producing restricted, microscopically discernible zones of erythrocyte destruction. The discrete nature of the plaques was presumably a result of repeated cycles of preferential invasion and lysis of immediately adjacent host cells upon completion of each erythrocytic growth cycle. To optimise conditions for plaque formation and detection, further assays were performed using a range of haematocrit values. This revealed that a haematocrit of 0.75% (~7.5 x 10^7^ erythrocytes per ml, or ~1.5 x 10^7^ cells per well) resulted in optimal plaque morphology and clarity (Fig 1D). Interestingly, erythrocyte longevity under the conditions of the assay was also haematocrit-dependent, with clear signs of generalised erythrocyte lysis (i.e. appearance of free haemoglobin in the medium) typically evident as early as 10 days at a 0.5% haematocrit, whilst at higher haematocrits the cells were reproducibly completely stable for at least 2 weeks. All subsequent work therefore used a haematocrit of 0.75%. Typically, plaques attained a maximum diameter of ~0.1 mm before eventual complete lysis of all the erythrocytes between 16–21 days. Note that this was not due to excessive parasitaemia in the microwell cultures; microscopic examination of Giemsa-stained cultures recovered from wells containing 30 plaques at 14 days revealed parasitaemia values of only ~0.06% (~1 parasite in 1600 erythrocytes examined).

### Each *P*. *falciparum*-Infected Erythrocyte in the Starting Population Forms One Plaque

We reasoned that during parasite growth in the environment of a microplate well, even under the static conditions described above, diffusive movement of free merozoites or even infected erythrocytes might allow multiple plaques to form from a single parental infected cell. To investigate this possibility, a synchronous parasite culture at ring-stage was diluted into a suspension of fresh erythrocytes at 0.75% haematocrit in order to obtain a theoretical density of <5 parasitised erythrocytes per ml of culture. The suspension was dispensed into the central wells of 4 flat-bottomed plates (200 μl/well, 240 wells total), then the resulting plaques enumerated 15 days later. If the parasitised cells assort randomly and if each parasite-infected cell produces only a single discrete plaque, the numerical distribution of plaques across the wells is expected to follow Poisson statistics. A key indicator of this is that the experimentally determined (observed) mean average number of plaques per well is sufficient to allow a prediction of the probability distribution of plaque numbers across all the wells. In the experiment depicted in Table 1, the observed frequency distribution of plaques per well was compared to the theoretical Poisson distribution with the same mean number of plaques per well as observed in this experiment (0.388). A chi-squared test of goodness-of-fit showed that the observed distribution of plaques per well was not significantly different from the expected Poisson distribution. Similar data were obtained from three additional independent plaque assays with widely varying mean values of plaque numbers per well; in each case, the observed distribution showed a good match with the expected Poisson distribution (S2 Fig). Together with the observed linear relationship between plaque number and parasite dilution, these results convincingly confirm that–at least under limiting dilution conditions where plaque numbers can be accurately counted–each plaque usually or always derives from a single precursor parasite-infected erythrocyte and multiple plaques do not frequently arise from a single infected cell.

**Table 1:** Poisson analysis of plaque formation shows that each plaque derives from a single parasite-infected erythrocyte.

	Plaque frequency	
Plaques/well	Observed^a^	Expected^c^
**0**	168	163
**1**	56^b^	63
**2**	11^b^	12
**3**	5^b^	2

^a^Plaque number in a total of 240 wells (4 microwell plates).

^b^The total number of plaques in the four plates was 93, so the overall mean number of plaques/well (λ) is given by 93/240 = 0.388.

^c^Expected values assuming a Poisson frequency distribution. A chi-squared goodness of fit comparison of the observed and expected data gave a value χ^2^ = 1.25 for 2 degrees of freedom, p>0.50, indicating no significant difference between the sets of values. Note that for the chi-squared calculations, data for the 2 plaques/well and 3 plaques/well were pooled in order to avoid using expected values of <5.

### Use of the Plaque Assay for Phenotypic Analysis of a Replication-Defective *P*. *falciparum* Mutant

**Fig 2:**
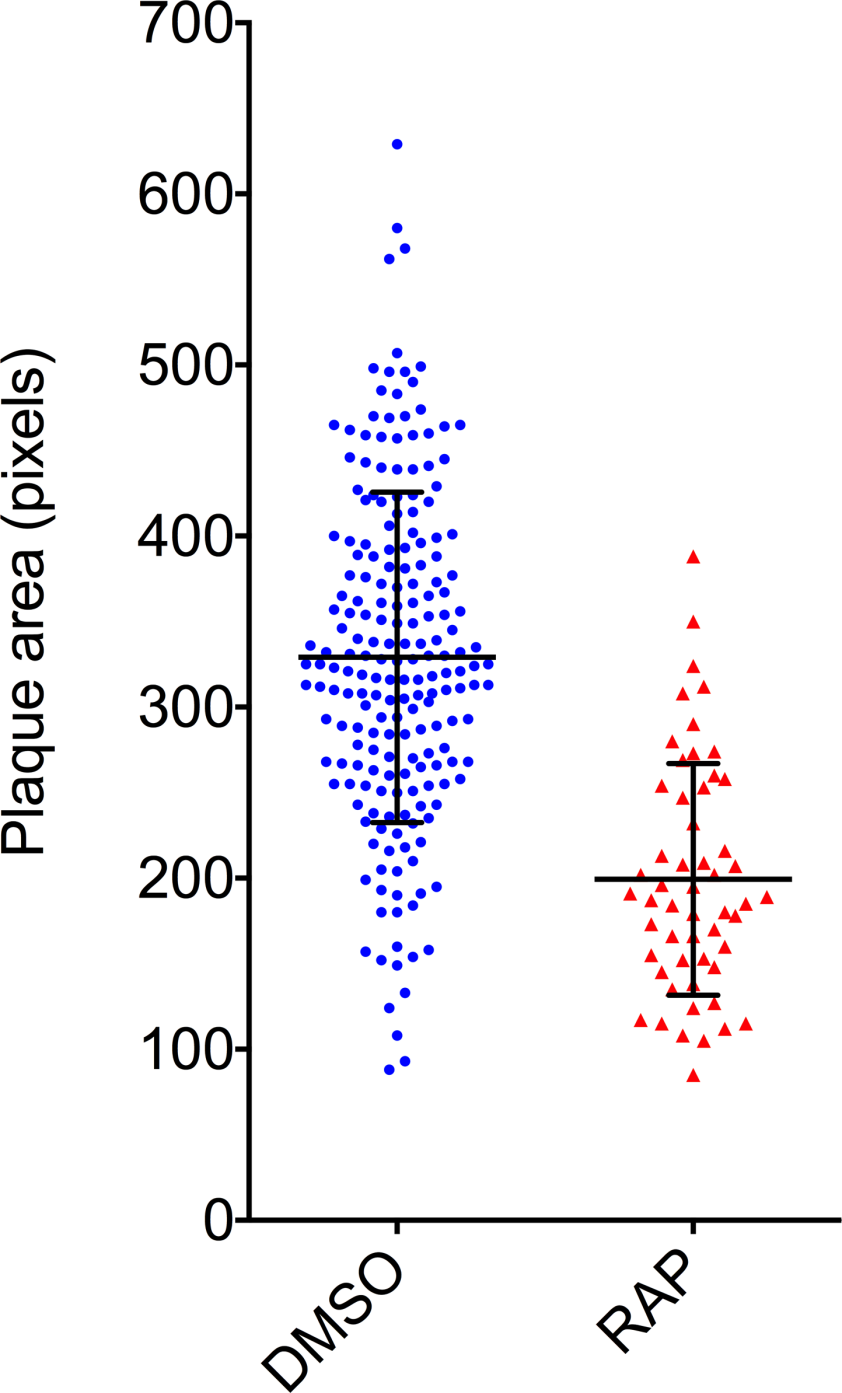
Phenotypic characterisation of an MSP1 mutant using the plaque assay. Scatter plots showing the distribution of plaque sizes obtained following treatment of MSP1:loxPint parasites with DMSO (control, mock-treated) or RAP to induce DiCre-mediated truncation of MSP1. Plaque numbers (n = 205 for the DMSO-treated samples and n = 54 for the RAP-treated samples) were counted manually. Plaque dimensions were quantified using the Magic Wand tool of Photoshop CS5 (Adobe). Horizontal bars indicate mean plaque area ± 1 SD.

To explore the broader utility of the plaque assay, we applied it to the phenotypic characterization of a genetically modified *P*. *falciparum* clone known to display a partially-defined growth defect. *Plasmodium* merozoites are uniformly coated with a glycolipid anchored surface protein called MSP1. We have previously shown [8] that conditional genetic truncation of the *P*. *falciparum MSP1* gene to remove its C-terminal membrane-anchoring domain results in merozoites that entirely lack surface-bound MSP1. These merozoites undergo highly inefficient egress from the host erythrocyte, with a resulting defect in parasite replication under static conditions *in vitro*. We predicted that this defect would manifest itself in the plaque assay as a reduction in the number and/or size of plaques. To test this prediction, a culture of highly synchronized ring-stage *P*. *falciparum* MSP1:loxPint parasites [8, 9], which possess a *loxP*-flanked 3’ segment of their *MSP1* gene and constitutively express a rapamycin (RAP)-inducible form of Cre recombinase (DiCre) [10], was divided into two. The cultures were then treated in parallel with either RAP (to induce DiCre-mediated gene excision) or DMSO (control solvent) only. The two treated cultures were washed, diluted identically to a theoretical ~75 parasitised cells per ml (15 parasites/well, 0.75% haematocrit), dispensed into flat-bottomed microplates and the resulting plaques examined 14 days later. Imaged plaque numbers were determined by manual counting, whilst the pixel area of individual plaques was quantified using the Magic Wand tool of Adobe Photoshop CS5. As shown in Fig 2, plaques formed in the RAP-treated cultures were smaller and less numerous (mean = 199.3, SD = 9.2, n = 54) than those formed in the control wells (mean = 329.1, SD = 6.7, n = 205), with a two-tailed independent t-test revealing this difference to be highly significant (t = 9.290, d.f. = 257, p<0.0001). The differences in mean plaque area values indicate a fitness defect associated with truncation of MSP1, consistent with the previously observed ~2-fold slower replication rate displayed by this mutant relative to the parental parasite clone in standard parasite growth assays [8]. Collectively, these results show that the plaque assay can be used as a simple means of rapidly characterizing even rather subtle effects of mutagenesis on parasite fitness.

### Rapid Phenotypic Analysis of a Conditional Lethal *P*. *falciparum* Mutant

**Fig 3:**
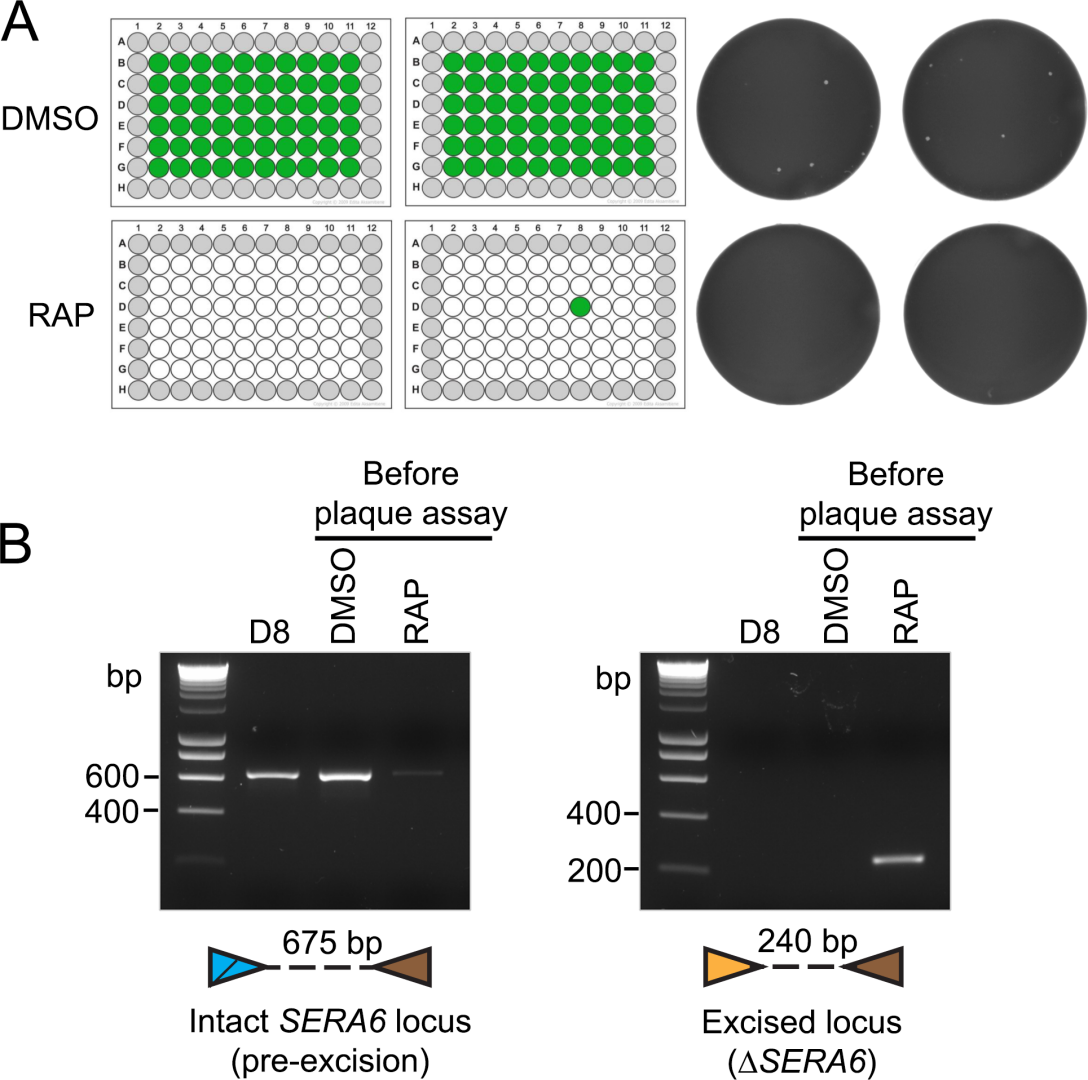
Rapid phenotypic characterization of a lethal conditional *P*. *falciparum* mutant using the plaque assay. (A) Left hand-side; schematic of the results of plaque analysis of RAP-treated and DMSO (mock)-treated SERA6:loxP parasites. Microplate wells coloured green indicate those that contained plaques 14 days following plating out the parasites at a theoretical 10 parasites/well. White wells contained no plaques (wells shown in grey were not used for the cloning). Whereas plaques were present in every well of the mock-treated culture, only a single plaque appeared in one well (well D8) of the RAP-treated culture. Right hand-side; example wells from the RAP-treated and control plates (green channel only of the scanned image shown to enhance plaque visibility, displayed as a grayscale image; see Materials and Methods for details). (B) Diagnostic PCR analysis of either the bulk SERA6:loxP parasite population immediately following RAP or DMSO-treatment (before plaque assay), or parasites expanded from well D8 of the +RAP plate. RAP-treatment significantly reduced the intact-*SERA6* locus-specific signal in the parasite population and resulted in appearance of a signal specific for the excised locus. Parasites rescued from well D8 of the RAP-treated parasites displayed a non-excised genomic architecture. The results strongly suggest that excision of the *SERA6* gene is lethal. Arrow-heads indicate the oligonucleotide primers used for PR analysis: blue, SERA6-34; yellow, JTS5synthF; brown, JTPbDT3’R (see Materials and Methods for primer sequences and PCR parameters). Expected sizes of the PCR amplicons are indicated.

To further examine the applicability of the plaque assay, we used it to examine a second *P*. *falciparum* mutant in which the DiCre system was used to conditionally disrupt a gene predicted to be essential for *in vitro* growth. The malarial serine-rich antigen (SERA) family comprises a group of papain-like proteins encoded by genes with variable numbers of orthologues in all *Plasmodium* species examined [11]. Interest in the SERA proteins, which all share similarities with papain-family proteases, has arisen from evidence that they play roles in parasite egress and/or host cell invasion. Previous attempted direct gene disruption experiments using targeted homologous recombination have suggested that two of the nine *P*. *falciparum SERA* genes, *SERA5* and *SERA6*, are essential in asexual blood stages [12, 13]. To extend our previous work indicating that *SERA6* may have a function at egress [14], we produced transgenic *P*. *falciparum* parasites (called SERA6:loxP) in which the entire *SERA6* locus was flanked by *loxP* sites (manuscript in preparation; J. Thomas, C. Collins and M. Blackman). These parasites were produced on the genetic background of the same DiCre-expressing parasite clone (called 1G5DC [10]) used to produce the MSP1:loxPint parasites described above. A synchronous culture of a SERA6:loxP clone was divided into two and treated in parallel with either RAP to induce DiCre-mediated gene excision, or DMSO only. The treated cultures were washed, diluted identically to a theoretical ~75 parasites per ml (15 parasites/well), dispensed into flat-bottomed microplates and the resulting plaques imaged 14 days later. As shown in Fig 3A, the results demonstrated a dramatic difference between the control DMSO-treated cultures, which showed substantial numbers of plaques, and the RAP-treated parasites, in which only a single plaque appeared in one well. RAP-mediated gene excision in the *P*. *falciparum* DiCre system, whilst tightly regulated and highly efficient, is rarely complete in a treated population [8, 10]. This can result in heterogeneous parasite populations that contain both excised and non-excised parasites, making it difficult to unambiguously assess the effects of gene modification on parasite fitness in uncloned RAP-treated cultures. Based on the clear difference between the RAP-treated and control SERA6:loxP parasites in the plaque assay, we hypothesized that the single plaque formed in the RAP-treated cultures might be derived from a parasite that had not undergone excision of the floxed *SERA6* gene. To test this, parasites from the plaque-positive well in the RAP-treated samples were expanded and analyzed by diagnostic PCR. This showed that these parasites indeed possessed an intact, non-excised genomic *SERA6* locus (Fig 3B). These results convincingly demonstrate that loss of SERA6 expression produces non-viable parasites that are completely unable to produce plaques. Importantly, because the plaque assay allowed visualization of parasite fitness at the level of individual parasite clones, it enabled simple comparison of the RAP-treated and control cultures and allowed us to quickly demonstrate that the only parasites in the RAP-treated population capable of surviving were those rare parasites in which excision of the *SERA6* gene had not occurred.

## Discussion

There has long been a need for a simple and robust plaque assay suitable for use with *P*. *falciparum*. Here we have developed such a system, using the simple expedient of culturing the parasite under static, limiting dilution conditions in human erythrocytes in flat-bottomed microplates. Visualisation of plaque formation was haematocrit-dependent, but we found that plaques were easily detectable within ~2 weeks under optimised conditions (0.75% haematocrit). Based on a mean human erythrocyte volume of ~90 x 10^−15^ L [15], we calculate that under the conditions used the settled erythrocytes formed layers that are at least 24 cells thick. This likely allows productive parasite proliferation and plaque growth in 3 dimensions, as opposed to the two dimensional plaque expansion allowed by the adherent erythrocyte monolayers used in previously-described *P*. *falciparum* plaque assays. The plaques, which presumably comprise predominantly haemoglobin-free erythrocyte ghosts, cell debris and haemozoin pigment, are completely stable under conditions of regular manual plate transferral to and from incubators, even without the use of semi-solid media, probably because the small volumes in each microplate well limit liquid movement and consequent disturbance of the erythrocyte layer. Use of larger wells or flasks would probably destabilise plaques, which may explain why the formation of these structures has not previously been described. Plaque integrity and stability was further ensured in the assay by not replacing the medium during the 2–3 week period of the assay. Although this may have the effect of limiting parasite growth rates, we found that up to a plaque density of at least ~340 plaques per well, plaque frequency correlated with starting parasite density, implying that—at least within this parasitaemia range—plaque formation is not adversely affected by plaque density. Plaques produced by clonal parasite populations were not completely homogeneous in size or shape, but this is a feature of many plaques, including those produced by *Toxoplasma*. The *P*. *falciparum* plaques are easily visible using an inverted light microscope and, similar to viral plaques [16], can be rapidly imaged with minimal disturbance using an inexpensive, commercially available document scanner, avoiding the need for laborious photographic imaging of individual wells. Indeed, using a high resolution (4,800 dpi) flat-bed scanner in transmission light mode, we found that we were able to scan and document up to 3 plates simultaneously in a single TIFF image, allowing for rapid documentation of multiple plates. The ready availability, low cost and lack of user training required to use a flat-bed scanner makes this a highly accessible, medium-throughput means of documenting the results of assays.

The *P*. *falciparum* plaque assay has important advantages compared to those commonly used for viruses and other intracellular pathogens such as *Toxoplasma*. Mature erythrocytes do not replicate, so there is no need to take into account changes in host cell confluency over the course of the assay. Furthermore, there is no requirement to fix and stain the cells before plaque analysis. The ability to easily visualise parasite clonal growth has other advantages. In our laboratory we now regularly use the approach for rapid limiting dilution cloning of *P*. *falciparum* lines without the need for laborious determination of starting parasitaemia values, simply by performing serial dilutions of a parasite culture across a plate then picking clones only from those wells that contain a single plaque. It is important to transfer these clones from the plaque assay plates into cultures containing fresh erythrocytes before the plaque assay cultures begin to undergo generalised erythrocyte lysis, which we found usually occurs between 16–21 days after initiating the plaque assay. The plaque assay may be applicable to the study of other *Plasmodium* species that can be cultivated *in vitro*, though our attempts to optimise it for use with culture-adapted lines of *P*. *knowlesi* [17] were not encouraging; in this case the plaques formed were irregular and indistinct, perhaps a result of the well-documented greater extracellular motility and longevity of free *P*. *knowlesi* merozoites [18, 19] which may allow more rapid dispersal of released merozoites within the wells than occurs with *P*. *falciparum* (unpublished data, J. Thomas).

Reverse genetic technologies have become increasingly important in malarial research over the past 2 decades. With the relatively recent introduction of Cas9-mediated mutagenesis [20, 21] and conditional gene modification technologies in *P*. *falciparum* [10, 22–24] we believe that the development of simple assays such as that described here is particularly timely. The plaque assay has multiple applications; here we have shown that it can be used to rapidly phenotype genetic mutants without the need for complex equipment or facilities (e.g. flow cytometry or spectrophotometry), but it also will be invaluable for the calculation of kill-curves with anti-parasite drugs and antibodies, for the selection of drug-resistant or fast-growing mutants, for facile comparison of parasite fitness under a range of growth conditions (e.g. in different defined media) and numerous other uses. Differences in parasite growth rate are presumably propagated over the course of the 4–7 cycles required to produce visible plaques, providing some advantages over traditional growth assays in that the cultures do not need to be passaged or fed. Importantly, the assay complements other widely used approaches for quantitation of parasite growth, including light microscopy, flow cytometry and assays based on expression of parasite proteins or enzymes [25, 26], incorporation of fluorescent dyes [27, 28] or radioactive hypoxanthine [29]. Although not explored here, the assay may be suitable for scaling up to high throughput image-based screens using high content microscopy. We believe the plaque assay will prove a valuable addition to the experimental tools available for studying this important pathogen.

## Materials and Methods

### *P*. *falciparum* Maintenance and Manipulation

Parasites (wild type clone 3D7, the DiCre-expressing clone 1G5DC [10], and MSP1:loxPint or SERA6:loxP parasites which were produced on the 1G5DC genetic background [8, 9]) were routinely cultured at 37°C in human erythrocytes at 1–4% haematocrit in RPMI 1640 (Life Technologies) supplemented with 2.3 gL^-1^ sodium bicarbonate, 4 gL^-1^ dextrose, 5.957 gL^-1^ HEPES, 0.05 gL^-1^ hypoxanthine, 0.5% (w/v) Albumax II, 0.025 gL^-1^ gentamycin sulphate, and 0.292 gL^-1^ L-glutamine (complete medium) in a low oxygen atmosphere as previously described [30, 31]. Parasitaemia was never allowed to exceed 10%. Human blood was obtained from anonymised donors through the UK National Blood Transfusion service and was used within 2 weeks of receipt. No ethical approval is required for its use. Routine microscopic examination of parasite growth was performed by fixing air-dried thin blood films with 100% methanol before staining with 10% Giemsa stain (VWR international) in 6.7 mM phosphate buffer, pH 7.1. For routine microscopic determination of parasitaemia values, at least 5,000 erythrocytes were examined. Haematocrit values were determined using a haemocytometer (Marienfield; 0.1 mm depth and 0.0025 mm^2^ area), assuming that 100% haematocrit equates to 1 x 10^10^ normocytes ml^-1^. For synchronisation, mature schizont stage parasites were isolated on cushions of 70% (v/v) Percoll (GE Healthcare) adjusted to isotonicity as described [32, 33]. Lysis of mature forms of the parasite to enrich for ring forms after invasion was performed by suspending parasites in 5% (w/v) D-sorbitol [32, 34].

### Generation of Transgenic *P*. *falciparum* Clones and PCR Analysis

Production of the *P*. *falciparum* MSP1:loxPint clone which possess a *loxP*-flanked 3’ segment of the *MSP1* gene and constitutively expresses a RAP-inducible form of Cre recombinase has been described [8, 9]. Production of the SERA6:loxP line was achieved by introducing a *loxP* site into the genome directly downstream of the *SERA6* locus in the 1G5DC clone, which already possesses a genomic *loxP* site downstream of the *SERA5* locus. Details of the approach used to generate the SERA6:loxP parasites will be provided separately (manuscript in preparation; J. Thomas, C. Collins and M. Blackman). When desired, transgenic parasites were treated with RAP (100 nM final concentration) or DMSO (control vehicle, 1% v/v) for 4 h, as described [10]. Genomic DNA was prepared from treated parasites for diagnostic PCR analysis as described [10]. The intact modified *SERA6* locus was amplified by PCR using oligonucleotide primers JTPbDT3’R (5’-TTACAGTTATAAATACAATCAATTGG-3’) plus SERA6-34 (5’- GTCCTGGAAGAAGAACGTTGCCGCCGCGAGACACAACACTGACTTCATG-3’). The excised locus (following DiCre-mediated recombination) was amplified using primers JTPbDT3’R plus JTS5synthF (5’- GAATGCTATTTCTGCTACGTG-3’). For diagnostic PCR analysis a Kappa2G Fast HotStart ReadyMix PCR Kit (Kappa Biosystems) was used. Thermal cycle conditions for amplification of the intact modified *SERA6* locus were: initial denaturation at 95°C for 60 sec, followed by 20 cycles of 95°C for 10 sec, 51°C for 10 sec and 72°C for 15 sec, with a final extension at 72°C for 60 sec. Thermal cycle conditions for amplification of the excised locus were: initial denaturation at 95°C for 60 sec, followed by 25 cycles of 95°C for 10 sec, 55°C for 10 sec and 72°C for 15 sec, with a final extension at 72°C for 60 sec.

### Imaging, Documentation and Quantitation of Plaque Formation

To allow plaque formation, synchronous parasite cultures at ring stage were diluted to the desired densities in complete medium with human erythrocytes at haematocrits between 0.5% and 2%, then dispensed into flat-bottomed 96 well microplates (Costar 3596, Corning NY, USA; 0.32 cm^2^ growth area per well, diameter 6.38 mm) using 200 μl culture per well. Plates were incubated in gassed, humidified sealed modular incubator chambers (Billups-Rothenberg, CA, USA). To limit evaporation, only the inner 60 wells of microplates were used for cultures whilst sterile phosphate buffered saline was added to the outer wells of plates. Plaque formation was assessed routinely by microscopic examination using a Nikon TMS inverted microscope (40x magnification). When desired, plaque formation was documented without opening the plates using a Perfection V750 Pro scanner (Epson) in top-down transmission light mode, saving images as 4,800 dpi RGB TIFF files. Usually 3 microplates were imaged simultaneously with the scanner. Plaque visibility was generally enhanced by using the 'Split channels' function of the Fiji distribution of ImageJ [35] to split the RGB TIFF file into red, green and blue data channels, then using the green channel image for quantification of plaque dimensions, since this provided maximum contrast between plaques and the surrounding erythrocyte layer. Plaques were counted by visual examination of the images and plaque size quantified using the Magic Wand tool in Adobe Photoshop CS5 using a tolerance setting value of 32. This tool delineates the perimeter of selected plaques and calculates plaque area in pixels. No area value was recorded for apparent plaques that could not be delineated by the tool. Statistical analysis (linear regression analysis by analysis of covariance and t-test) was performed using GraphPad Prism 7 software (CA, USA).

## Supporting Information

S1 Fig The experimentally observed frequency distribution of plaques in the plaque assay follows a Poisson distribution. (PDF)

S2 Fig Plaque frequency shows a linear correlation with parasite number. (PDF)
