# Supplementary material for: Development and Application of a Simple Plaque Assay for the Human Malaria Parasite Plasmodium falciparum
Source: PLoS One. 2016 Jun 22;11(6):e0157873. doi: 10.1371/journal.pone.0157873 (PMC4917082; doi:10.1371/journal.pone.0157873)
Supplement: S1 Fig — (PDF) [file pone.0157873.s001.pdf]

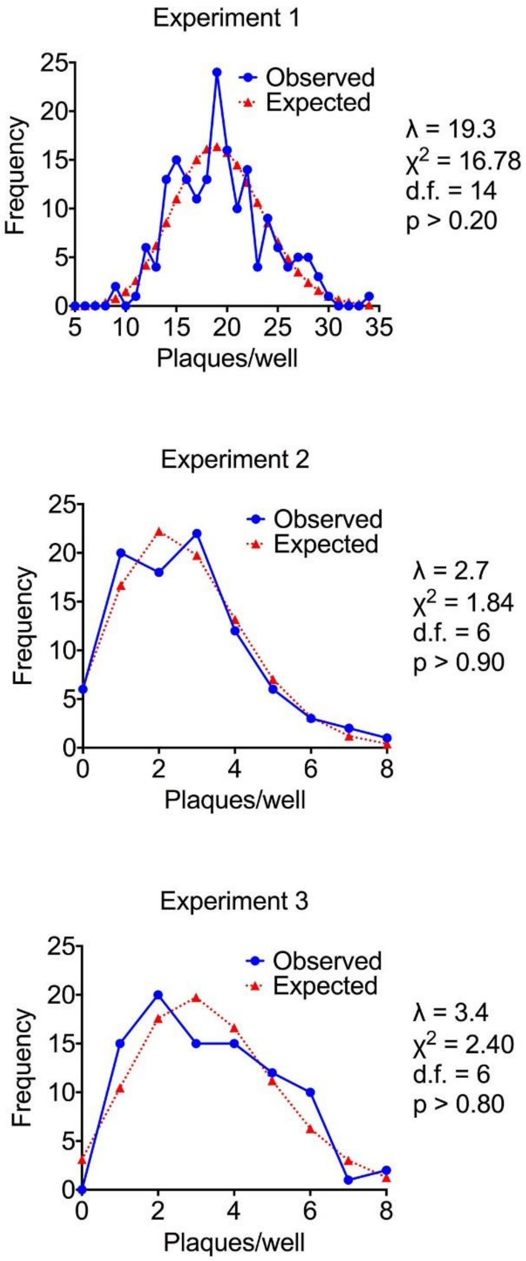

S1 Fig. The experimentally observed frequency distribution of plaques in the plaque assay follows a Poisson distribution. Depicted are plaque assay data from three independent experiments carried out on different days in which serially diluted 3D7 *P. falciparum* cultures (0.75% haematocrit) were cultured in flat-bottomed 96-well microplates as described in Materials and Methods. Plaques were enumerated at 14 days and mean values (λ) calculated. Experimentally observed plaque frequency values are shown plotted (blue) alongside the expected frequency values of a Poisson distribution with the same mean value (red). Chi-squared goodness-of-fit tests showed no significant deviation of observed distributions from the expected distributions. Chi-squared (χ^2) values, number of degrees of freedom (d.f) and p-values are indicated in each case. Note that for the purpose of statistical analysis, those categories where expected values were less than 5 were pooled to create a single category.
