# Supplementary material for: Development and Application of a Simple Plaque Assay for the Human Malaria Parasite Plasmodium falciparum
Source: PLoS One. 2016 Jun 22;11(6):e0157873. doi: 10.1371/journal.pone.0157873 (PMC4917082; doi:10.1371/journal.pone.0157873)
Supplement: S2 Fig — (PDF) [file pone.0157873.s002.pdf]

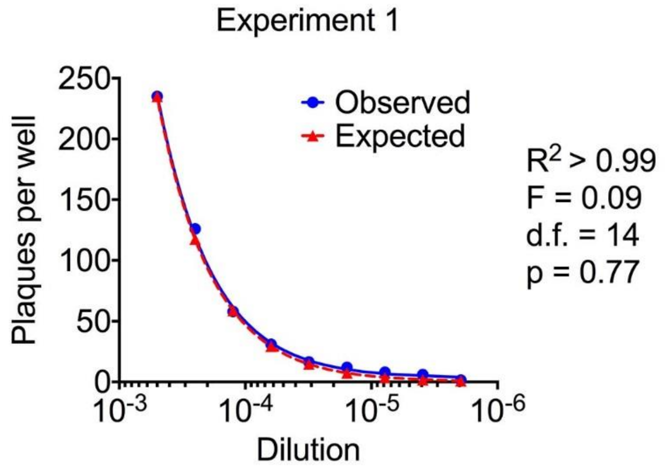

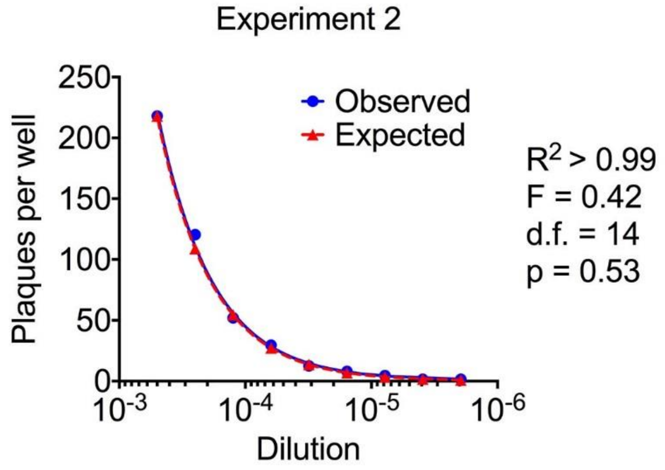

S2 Fig. Plaque frequency shows a linear correlation with parasite number. Depicted are plaque assay data from two independent experiments carried out on different days in which 2-fold serial dilutions of 3D7 *P. falciparum* cultures (0.75% haematocrit) were cultured in flat-bottomed 96-well microplates as described in Materials and Methods. Plaques were enumerated at 14 days. Plots of observed mean plaque frequency against dilution (blue) are shown alongside plots of plaque frequencies expected if there is a linear inverse correlation between plaque number and dilution (red). To establish the fit between the plots, linear regression was performed by analysis of covariance (ANCOVA) in GraphPad Prism. The results indicate a strong linear inverse correlation between dilution and plaque density in the assay, with no significant difference between the observed and expected curves. Values of the statistical data (R^2, F statistics, number of degrees of freedom and p values) are shown.
